# Virtual bracket removal: a comparative assessment of different software packages

**DOI:** 10.1186/s12903-024-04522-0

**Published:** 2024-07-10

**Authors:** Heba Gamil, Eiman Marzouk, Abbas Zaher

**Affiliations:** 1 Department of Orthodontics, Faculty of Dentistry, Alexandria University, Champollion St, P. O. Box: 21521, Azarita, Alexandria Egypt; 2 Department of Orthodontics, Faculty of Dentistry, Alexandria University, Alexandria, Egypt

**Keywords:** Virtual bracket removal, Intra-oral scans, Dental models, Orthodontic brackets, Computer aided design, 3Shape OrthoAnalyzer, Meshmixer, EasyRx, Medit Link

## Abstract

**Background:**

High precision intra-oral scans, coupled with advanced software, enable virtual bracket removal (VBR) from digital models. VBR allows the delivery of retainers and clear aligners promptly following debonding, thus reducing the patients' appointments and minimizing the likelihood of tooth movement. The objective of this study was to compare the enamel surface before bonding and after VBR using three different Computer-aided design (CAD) software and to compare their accuracy.

**Methods:**

Maxillary scans of 20 participants starting orthodontic treatment were selected for inclusion in the study, who exhibited mild to moderate crowding and required bonding of brackets on the labial surface of permanent maxillary teeth (from the maxillary left first molar to the maxillary right first molar). Two intra-oral scans were conducted on the same day, before bonding and immediately after bonding using CEREC Omnicam (Sirona Dental Systems, Bensheim, Germany). The virtual removal of the brackets from the post-bonding models was performed using OrthoAnalyzer (3Shape, Copenhagen, Denmark), Meshmixer (Autodesk, San Rafael, Calif, USA), and EasyRx (LLC, Atlanta, GA, USA) software. The models that underwent VBR were superimposed on the pre-bonding models by Medit Link App (Medit, Seoul, South Korea) using surface-based registration. The changes in the enamel surface following VBR using the three software packages were quantified using the Medit Link App.

**Results:**

There was a significant difference among the 3Shape, Meshmixer, and EasyRx software in tooth surface change following VBR. Specifically, EasyRx exhibited lower levels of accuracy compared to the other two VBR software programs (*p*<.001, *p*<.001). A significant difference in enamel surface change was observed between tooth segments across all software groups, in both incisors and molars, with VBR of the molars exhibiting the lowest level of accuracy (3Shape *p*=.002, Meshmixer *p*<.001, EasyRx *p*<.001). Regarding the direction of tooth surface changes following VBR, it was observed that all three groups exhibited a significant increase in the percentage of inadequate bracket removal across all teeth segments.

**Conclusions:**

3Shape and Meshmixer manual VBR software were found to be more accurate than EasyRx automated software, however, the differences were minimal and clinically insignificant.

## Background

The integration of technological advancements in orthodontic offices has revolutionized the process of diagnosis and treatment planning. The implementation of digital technology has resulted in enhanced accuracy, efficiency, consistency, and predictability of treatment outcomes [1].

Prompt delivery of retainers following the removal of brackets or attachments is important to avoid unwanted tooth movement [2, 3]. Conventionally, the workflow for the fabrication of retainers follows one of two methods: direct or indirect. In the direct approach, an impression is taken after the removal of the brackets, the impression is poured in stone, and then the retainer is fabricated using the stone model [4]. In the indirect approach, an impression is taken prior to the bracket removal, and it is poured in stone. Then the dental lab personnel physically carve off the brackets from the stone model before proceeding with the fabrication of the retainer [5].

Additionally, hybrid orthodontic treatment using clear aligners combined with fixed orthodontic appliances commonly necessitates the timely removal of the brackets at the time of insertion of the aligners [6, 7].

The manufacturing processes for both retainers and clear aligners often involve several days, and it is possible for complications to occur that need the patient to return to the dental office for a new impression [5]. Dental relapse has the potential to manifest shortly after the removal of orthodontic appliances, leading to the development of gaps or alterations in the alignment of teeth [2, 3]. The insertion of retainers or clear aligners after relapse might result in significant pressure being applied to teeth that have undergone positional changes, potentially leading to tooth instability and apical root resorption [2].

The fabrication techniques for orthodontic appliances have seen significant advancements with the emergence of intraoral scanners, computer-aided design (CAD), and computer-aided manufacturing (CAM) software [8, 9].

One such breakthrough is the possibility of virtual removal of brackets from the digital models, which allows the delivery of retainers or aligners immediately following debonding.

The production of orthodontic appliances using this approach involves acquiring an intra-oral scan of the patient's teeth and processing the resulting digital models in the stereolithography (STL) file format. Subsequently, a virtual bracket removal (VBR) procedure is performed using CAD software, as proposed by Marsh et al. [5].

Various CAD software programs have been developed that can perform VBR. Examples of such programs include OrthoAnalyzer (3Shape, Copenhagen, Denmark) [10] and Meshmixer (Autodesk, San Rafael, Calif, USA) [11] which rely on the expertise of the operator to manually remove brackets. On the other hand, EasyRx software (LLC, Atlanta, GA, USA) [12], an internet-based platform that provides automated VBR services for a fee, employs artificial intelligence (AI) to automate the process of bracket removal.

Nevertheless, A search through the published literature revealed a lack of research that compared the accuracy of the three software programs.

Hence, the current study aimed to assess the enamel surface change before bonding and after virtual bracket removal using 3Shape OrthoAnalyzer, Meshmixer, and EasyRx software, and to compare the accuracy of the three software.

The null hypothesis was that there was no difference in the enamel surface before bonding and after virtual bracket removal using the three software packages.

## Materials and methods

This Diagnostic accuracy study was conducted according to the STARD guidelines [13] to assess the difference between the enamel surface before bonding and after virtual bracket removal using three different CAD software, and to compare between them. The institutional review board of Alexandria University’s Faculty of Dentistry approved this study (IORG:0008839-IRB:00010556), with serial number: 0409-03/2022. Informed consent was obtained from the patients or the legal guardians if the patients were under 18 years old.

### Sample size calculation

Based on Marsh et al. [5] adopting a statistical power (1 – β) of 80%, assuming a significance level of 95% (α=0.05), the minimum required sample size to conduct this accuracy study was found to be 19 patients. The sample size was calculated using Medcalc version 14.8.1. [14].

### Selection of the cases and acquisition of the scans

The study sample comprised maxillary arch scans of 20 individuals starting orthodontic therapy. Patients were included if they exhibited mild to moderate crowding and if their treatment plan involved bonding of brackets on the labial surface of permanent maxillary teeth from the maxillary left first molar to the maxillary right first molar.

Patients were excluded if they had missing teeth other than the second and third molars or if their treatment plan necessitated banding of the maxillary first permanent molars. Additionally, patients who had previous fixed orthodontic treatment with bonded appliances or had gingival hyperplasia were excluded.

Two intra-oral scans of the maxillary arch were obtained for each patient using CEREC Omnicam intra-oral scanner (Sirona Dental Systems, Bensheim, Germany). The two scans were performed in the same session: one before and one immediately after bonding.

The pre-bonding and post-bonding scans were exported as STL files and imported into the three tested software.

Upon importing the digital models into each software in STL format, the models were prepared by digitally eliminating any scanning artifacts related to the bracket surface. Artifacts in the 3Shape OrthoAnalyzer software were eliminated using the "Remove Artifacts" tool (0.150 mm). On the other hand, the artifacts were deleted in Meshmixer software by employing the "Brush" selection mode.

### Superimposition protocol

To ensure scanning accuracy, the Medit Design App, a component of the Medit Link software (Medit, Seoul, South Korea) [15], was used to superimpose the pre-bonding and the post-bonding models using the surface-based registration technique [16, 17].

To perform the superimposition, first, the alignment mode was employed by utilizing the "Align selected areas" tool. This tool enabled the selection of corresponding regions in the target data (post-bonding model) and the reference data (pre-bonding model) to align them together.

**Fig. 1:**
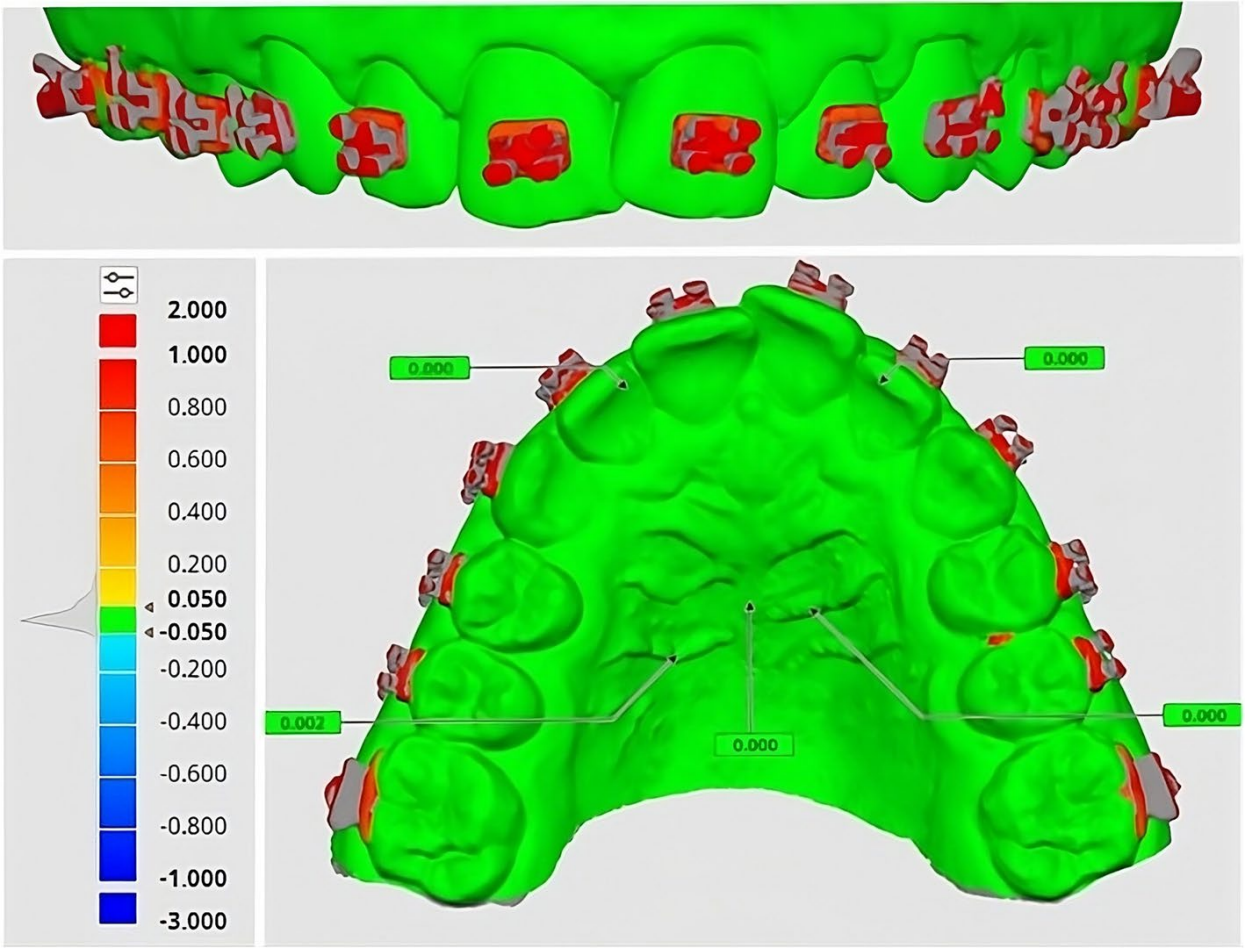
Superimposition of bonded models onto control using Medit Link software, superimposition accuracy confirmation using color-coded maps (green hue indicates surface changes ≤ 0.05 mm; blue or red hues indicate surface changes > 0.05 mm in different directions)

Afterwards, the "Deviation display mode" was employed to visualize the deviation results between the two datasets (Fig. 1). The deviation color bar was used to validate the superimposition accuracy on the regions unaffected by the VBR.

The quantification of errors in the fixed region (green color) where changes were not anticipated involved both eye examination and clicking on the model, this showed the exact numerical value in this area. The accuracy of superimposition was deemed satisfactory when the error ranged from 0 to 0.05 mm.

### Virtual bracket removal protocol

The 3Shape and Meshmixer VBR protocol utilized in this study was based on the established and verified method developed by Chamberlain-Umanoff [18].

**Fig. 2:**
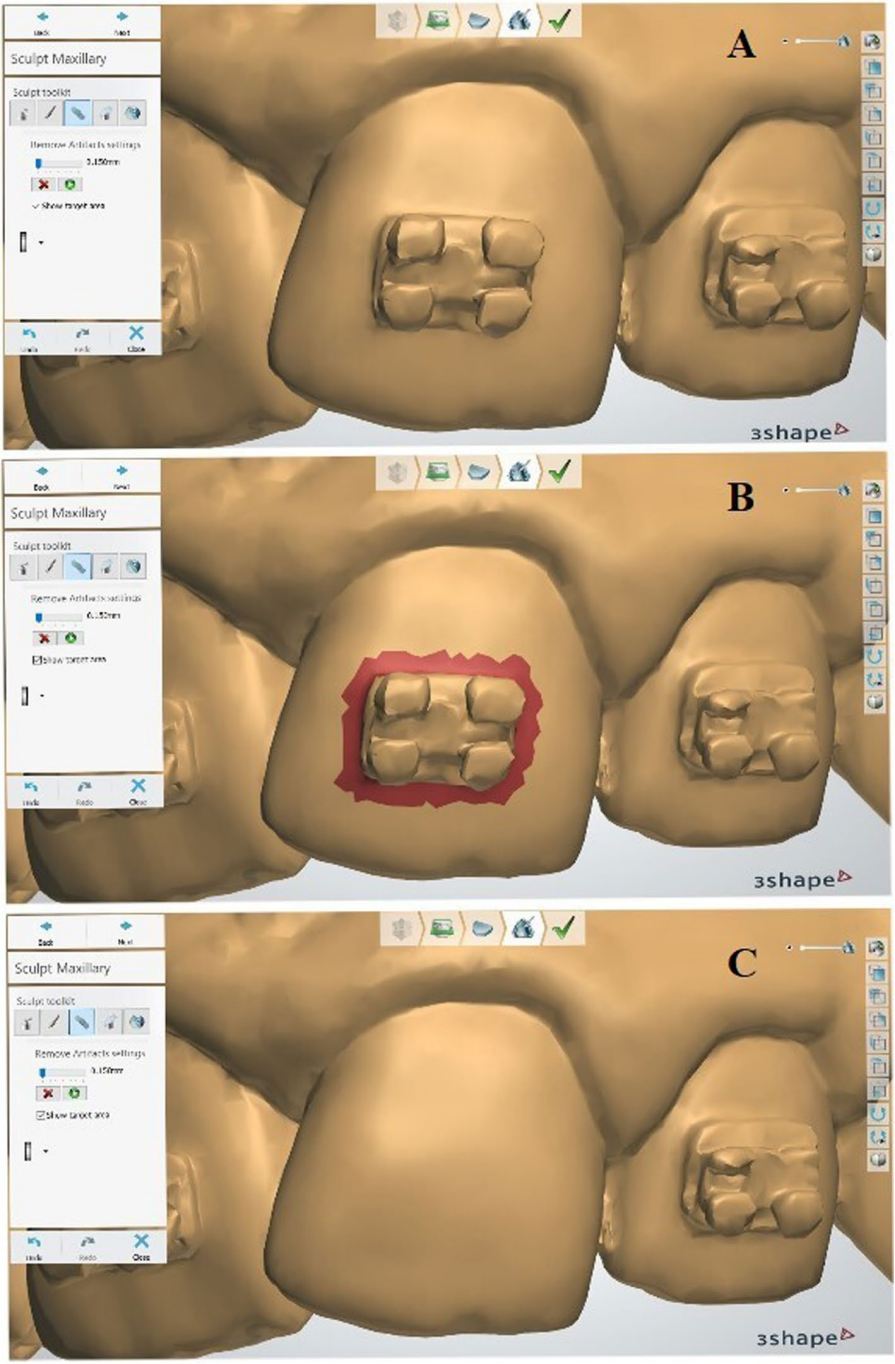
VBR with 3Shape OrthoAnalyzer; **A** Initial model with brackets; **B** Bracket selection using Remove Artifacts tool (0.150 mm); **C** Tooth after VBR

**Fig. 3:**
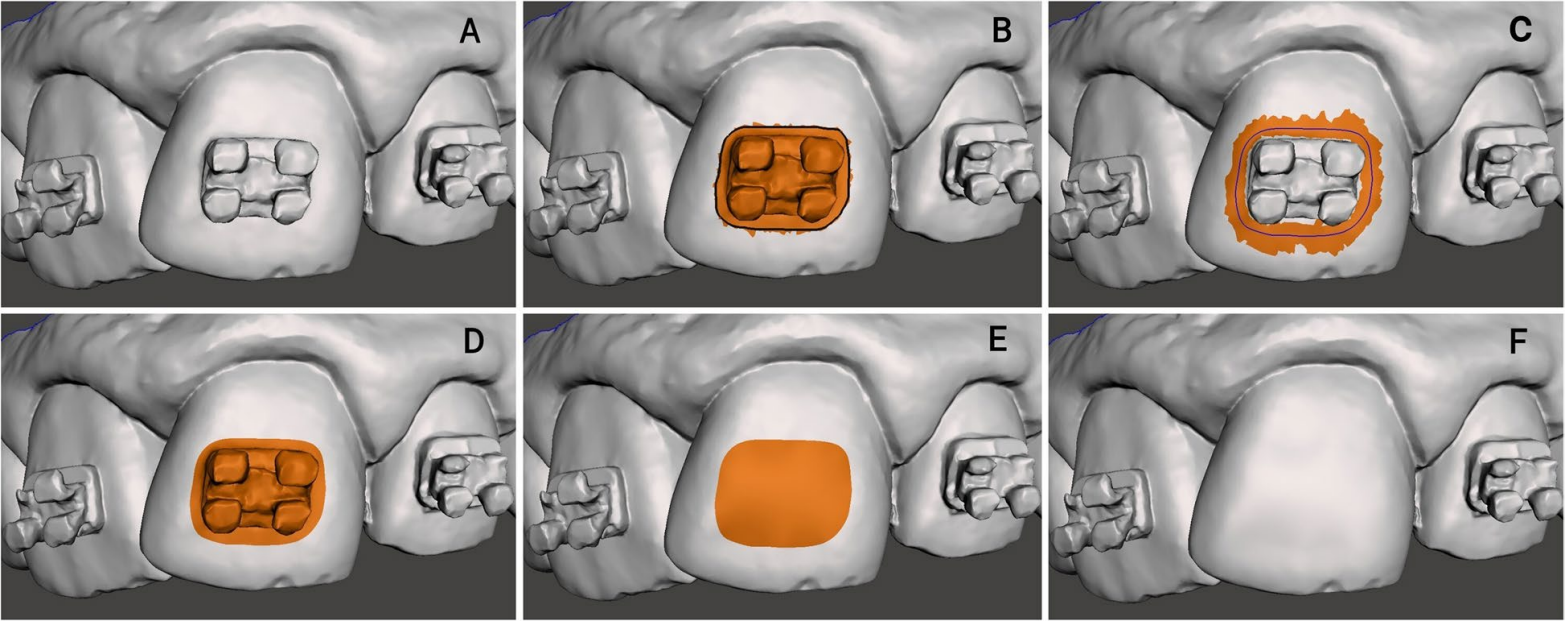
VBR with Meshmixer; **A** Initial model with brackets; **B** Bracket selection using Surface Lasso tool; **C** Using Smooth Boundary tool; **D** Refining the irregular selection boundary around the bracket; **E** Using the erase and fill tool to remove the bracket; **F** Tooth after VBR

In 3Shape OrthoAnalyzer software, the "Remove Artifacts" tool (0.150 mm) was utilized to accurately circumscribe the bracket as shown in (Fig. 2). While in Meshmixer, the bracket was delineated using the "Surface Lasso" selection mode. Subsequently, the selection boundaries were adjusted using the "Smooth Boundary" tool. The "Erase & Fill" tool was then used to virtually remove the bracket (Fig. 3). The same procedure was followed for all the maxillary teeth commencing at the right first molar and concluding at the left first molar.

**Fig. 4:**
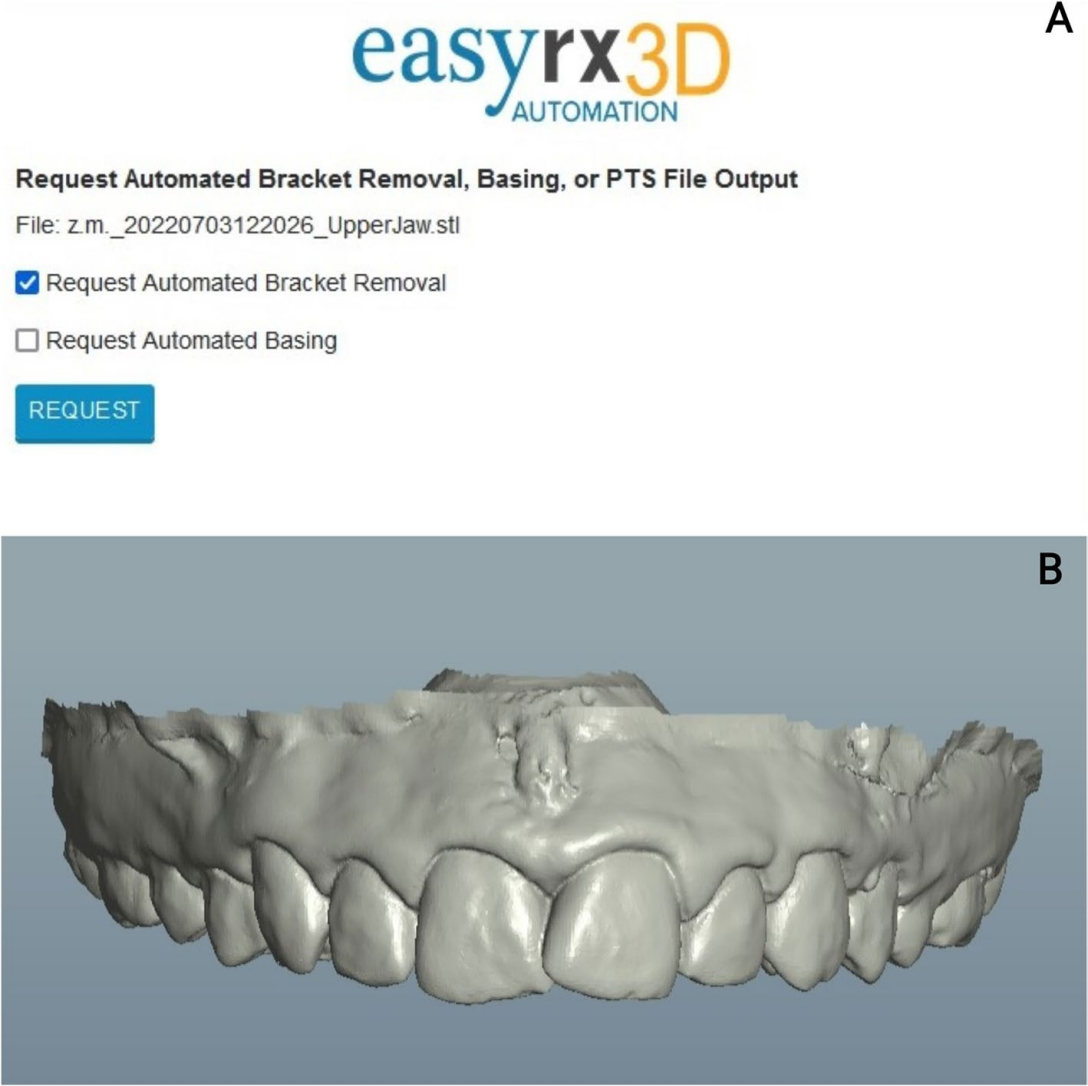
Automated VBR with EasyRx; **A** Request automated bracket removal; **B** Digital model after VBR

To perform VBR in EasyRx, the digital model was uploaded and the option to "Request Automated Bracket Removal" was chosen. After that, the digital model underwent processing, resulting in the acquisition of a bracket-free digital model (Fig. 4).

The post-VBR digital models were assigned to one of three groups according to the software used to perform the VBR.

- Group 1: 3Shape OrthoAnalyzer manual bracket removal software.
- Group 2: Meshmixer manual bracket removal software.
- Group 3: EasyRx automated bracket removal software.

To guarantee blinding, the three groups were assigned the labels A, B, and C by the principal researcher. The examiner responsible for performing the superimposition and measurements on the digital models was unaware of the specific software employed since it was concealed until the completion of the statistical analysis.

### Model preparation

**Fig. 5:**
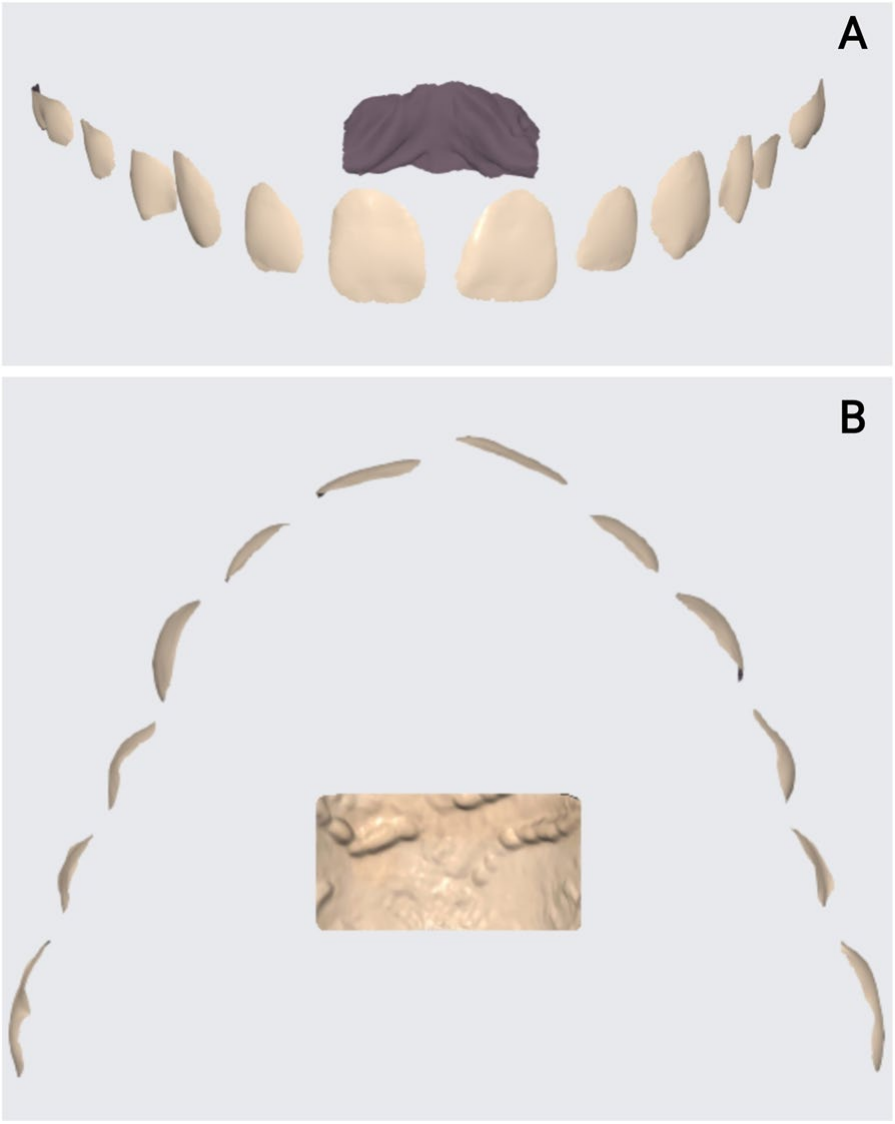
The trimmed model after using the Trimming tool in Medit Link software to include: **A** The facial surface starting 1 mm away from the gingival margin, incisal edges, mesial and distal line angles; **B** Part of the palate extending from the medial two-thirds of the third rugae and the area 5 mm dorsal to them

Following VBR and before measuring the enamel surface changes, the models were cropped to keep the labial surface of the teeth and part of the palate. The process started with one of the models in group A, where the "Trimming" tool was used to keep only the facial surface starting 1 mm away from the gingival margin, incisal edges, and mesial and distal line angles. Additionally, a portion of the palate was included to aid in superimposition extending from the medial two-thirds of the third rugae to the area 5 mm dorsal to them [19, 20] (Fig. 5).

The two models of the same patient in groups B and C were precisely adjusted to match the dimensions of the already trimmed model (group A) to ensure consistency in the collected data, this was done by superimposing the trimmed model and the untrimmed models.

### Measurement protocol

**Fig. 6:**
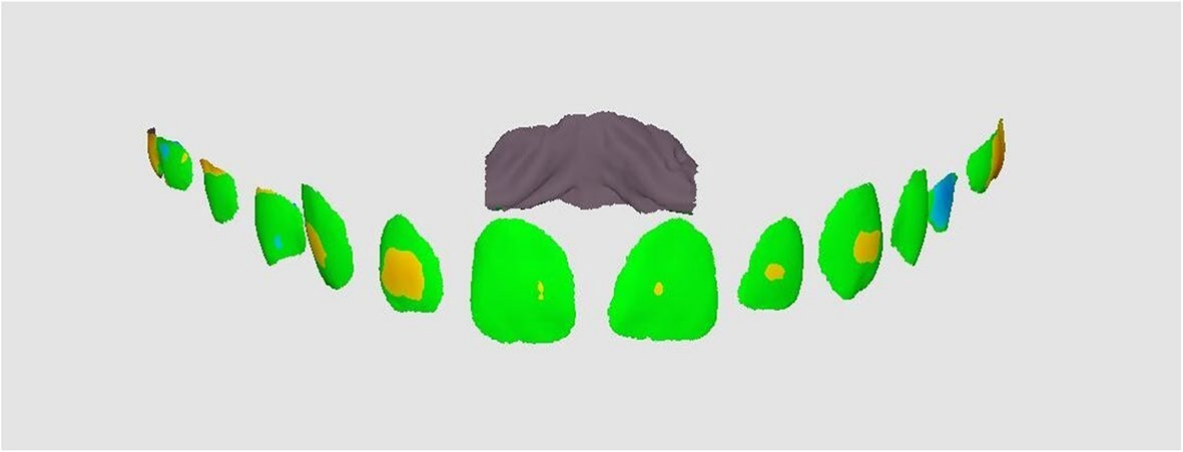
Evaluation of VBR using Medit Link software by the superimposition of the model after VBR on the Pre-bonding model

The pre-bonding model and the trimmed model after VBR for each patient were aligned using the Alignment mode. The deviation display mode was employed to automatically measure the linear changes on the facial surface to obtain the root mean square (RMS), which quantified the difference between the pre-bonding model and the model after VBR (Fig. 6). Additionally, to measure the deviation in the different tooth types, the trimmed models were divided into segments, namely incisors, canines/premolars, and molars. The measurements of deviation were then obtained for each segment separately in a manner consistent with the technique employed for the overall model.

The direction of surface change, whether excess or deficiency, relative to the prebonding models was determined using the deviation color bar. Red hues were indicative of positive values, which corresponded to regions where bracket removal was insufficient. On the other hand, blue hues represented negative values, indicating areas where unintended removal of tooth surface occurred. The aforementioned process was carried out for the three groups to be investigated.

Measurements of the digital models' superimposition of the entire sample were conducted again after a period of two weeks to evaluate the level of intra-examiner reliability, which was calculated using Kappa statistic [21]

### Statistical methodology

Normality was checked using descriptive statistics, plots, and normality tests. All data showed a non-normal distribution, so non-parametric analyses were adopted. Comparisons of RMS between the three studied software, and between different segments in each software used were performed using the Friedman test, followed by multiple pairwise comparisons using Bonferroni adjusted significance level. The chi-square test was used for comparisons of positive and negative tooth surface change. The significance level was set at *p*-value <0.05. Data were analyzed using IBM SPSS for Windows (Version 26.0, IBM Corp) [22].

## Results

Intra-examiner reliability was deemed excellent, with a value >0.9. The RMS values of surface changes were calculated for each segment and the overall model and were averaged for each software. Among the segments, the incisors exhibited the least amount of surface change, while the molars showed the greatest amount of surface change as a result of VBR (Table 1).

**Table 1:** RMS of the tooth surface changes after VBR using different software

		**3Shape (*n*=20)**	**Meshmixer (*n*=20)**	**EasyRx (*n*=20)**	***P* value 1**
**Incisors**	**Mean (SD)**	0.071 (0.015)	0.061 (0.016)	0.074 (0.020)	χ^r2^= 11.17 P= 0.004*
	**Median (IQR)**	0.068 (0.058, 0.084)	0.057 (0.049, 0.070)	0.072 (0.060, 0.086)	
**Canines/ premolars**	**Mean (SD)**	0.083 (0.025)	0.084 (0.035)	0.169 (0.124)	χ^r2^= 22.30 P <0.001*
	**Median (IQR)**	0.075 (0.063, 0.097)	0.076 (0.065, 0.094)	0.101 (0.088, 0.239)	
**Molars**	**Mean (SD)**	0.111 (0.036)	0.140 (0.053)	0.240 (0.183)	χ^r2^= 20.80 P <0.001*
	**Median (IQR)**	0.099 (0.093, 0.130)	0.127 (0.101, 0.182)	0.162 (0.121, 0.303)	
**Overall**	**Mean (SD)**	0.112 (0.021)	0.120 (0.037)	0.235 (0.160)	χ^r2^= 22.05 P <0.001*
	**Median (IQR)**	0.106 (0.093, 0.128)	0.114 (0.089, 0.146)	0.177 (0.129, 0.279)	
**P value 2**		χ^r2^= 15.18 P <0.001*	χ^r2^= 30.70 P <0.001*	χ^r2^= 30.70 P <0.001*	
**Post-hoc comparisons**	**Incisors vs. canines and premolars** **Incisors vs. molars** **Canines and premolars vs. molars**	1.00 0.002** 0.005**	0.008* <0.001** 0.03**	0.008** <0.001** 0.03**	

*SD* Standard Deviation, *IQR* Interquartile range

P value 1: Comparison between the three study groups, P value 2: comparison between different segments within each group

χ^r2^: Friedman test was used.

^*^Statistically significant at *p* value <0.05

^**^statistically significant differences between different segments *within* each group after Bonferroni adjustment

In 3Shape software, the Pairwise comparison of the RMS showed that RMS was significantly higher in molars compared with incisors and canines/premolars (*p*=.002, and *p*=.005, respectively) (Table 1). In Meshmixer software, the Pairwise comparison of the RMS of incisors was significantly lower compared with canines/premolars and molars (*p*=.008 and *p*<.001, respectively), the RMS of molars was significantly higher compared with canines/premolars (*p*=.03) (Table 1). Regarding the EasyRx software, the Pairwise comparison of the RMS of incisors was significantly lower compared with canines/premolars and molars (*p*=.008 and *p*<.001, respectively), the RMS of molars was significantly higher compared with canines/premolars (*p*=.03) (Table 1).

**Fig. 7:**
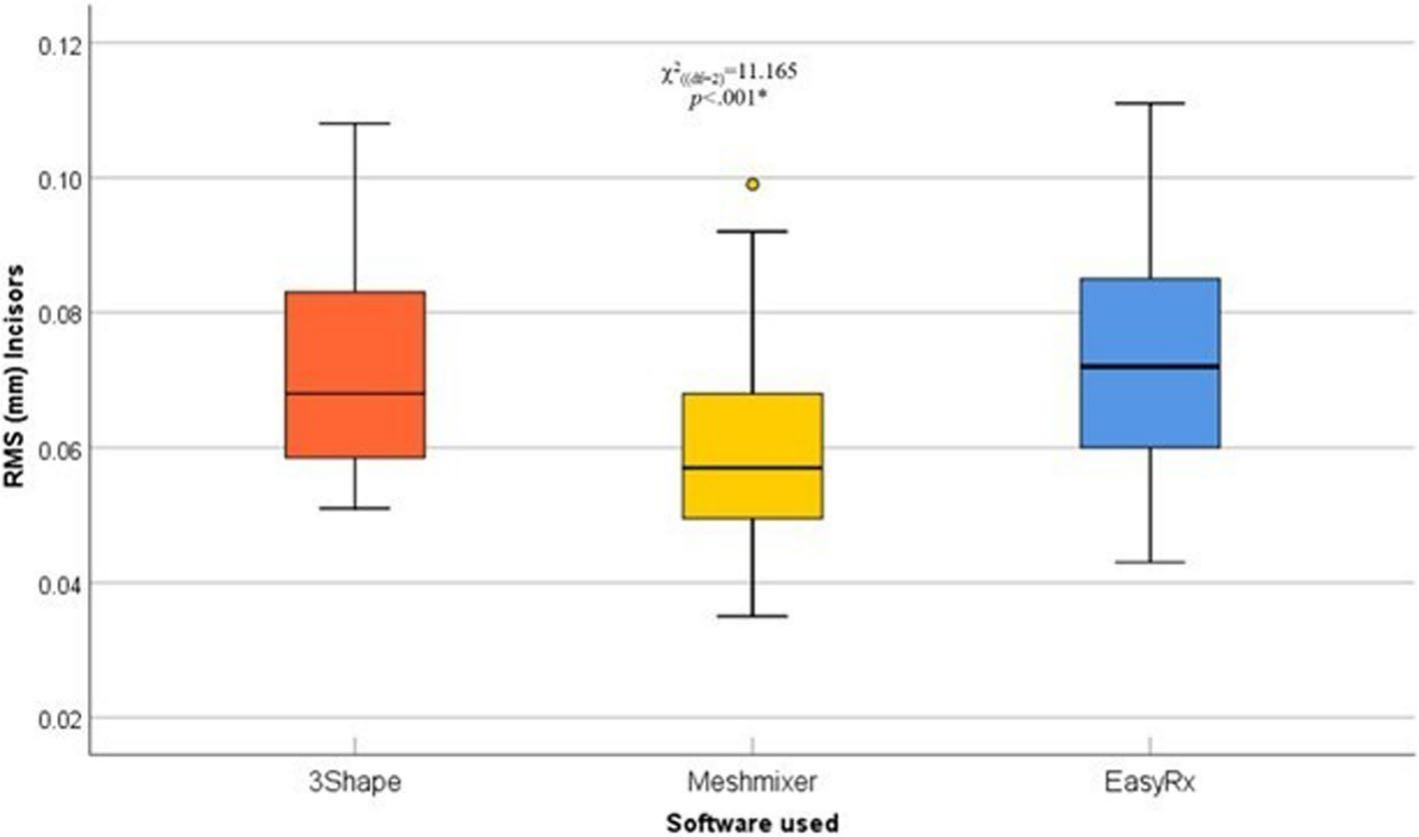
Box and whisker graph comparing RMS (mm) values in incisors in the three different software, the thick line in the middle of the box represents the median, the box represents the inter-quartile range (from 25^th^ to 75^th^ percentiles), the whiskers represent the minimum and maximum

**Fig. 8:**
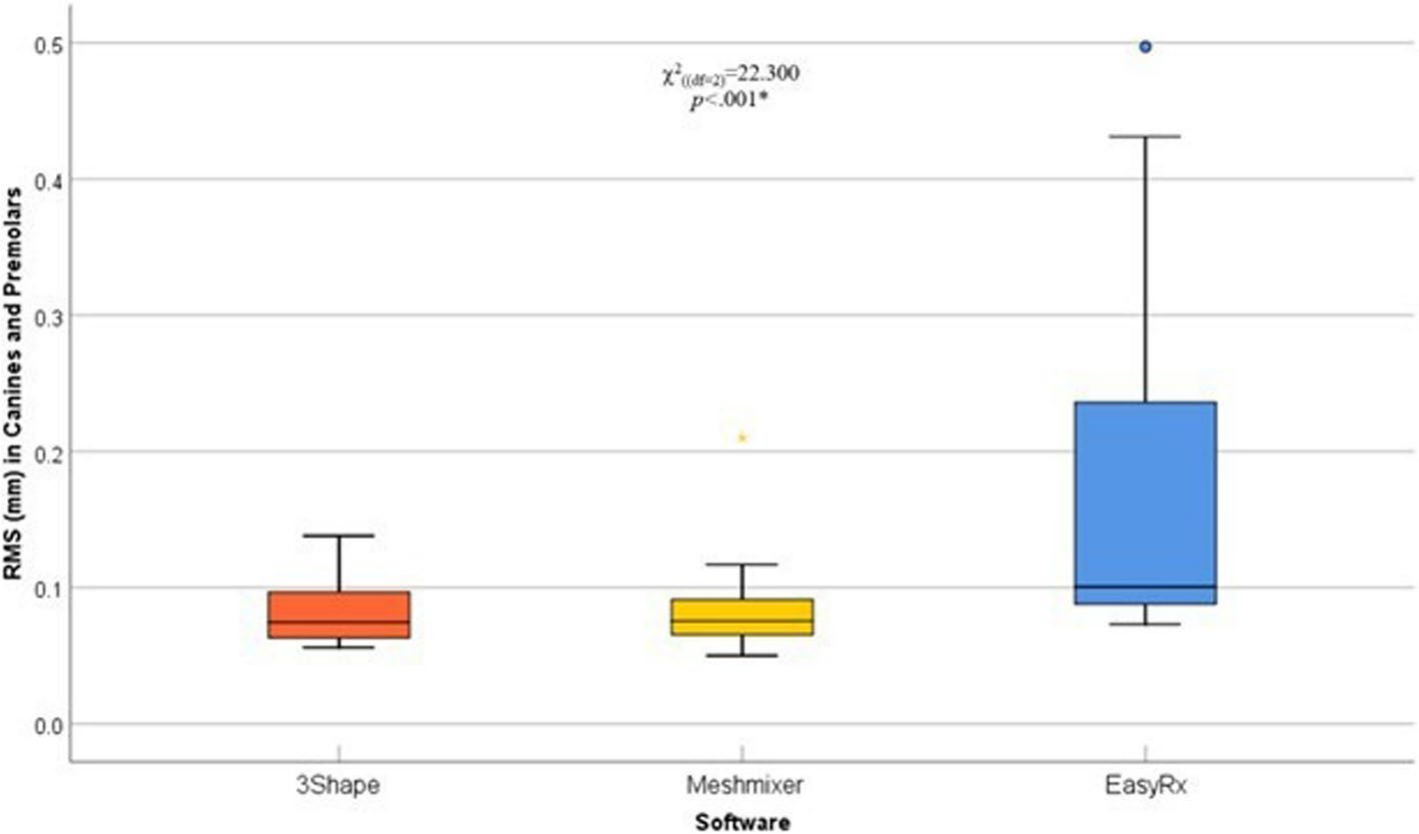
Box and whisker graph comparing RMS (mm) values in canines and premolars in the three different software, the thick line in the middle of the box represents the median, the box represents the inter-quartile range (from 25^th^ to 75^th^ percentiles), the whiskers represent the minimum and maximum

**Fig. 9:**
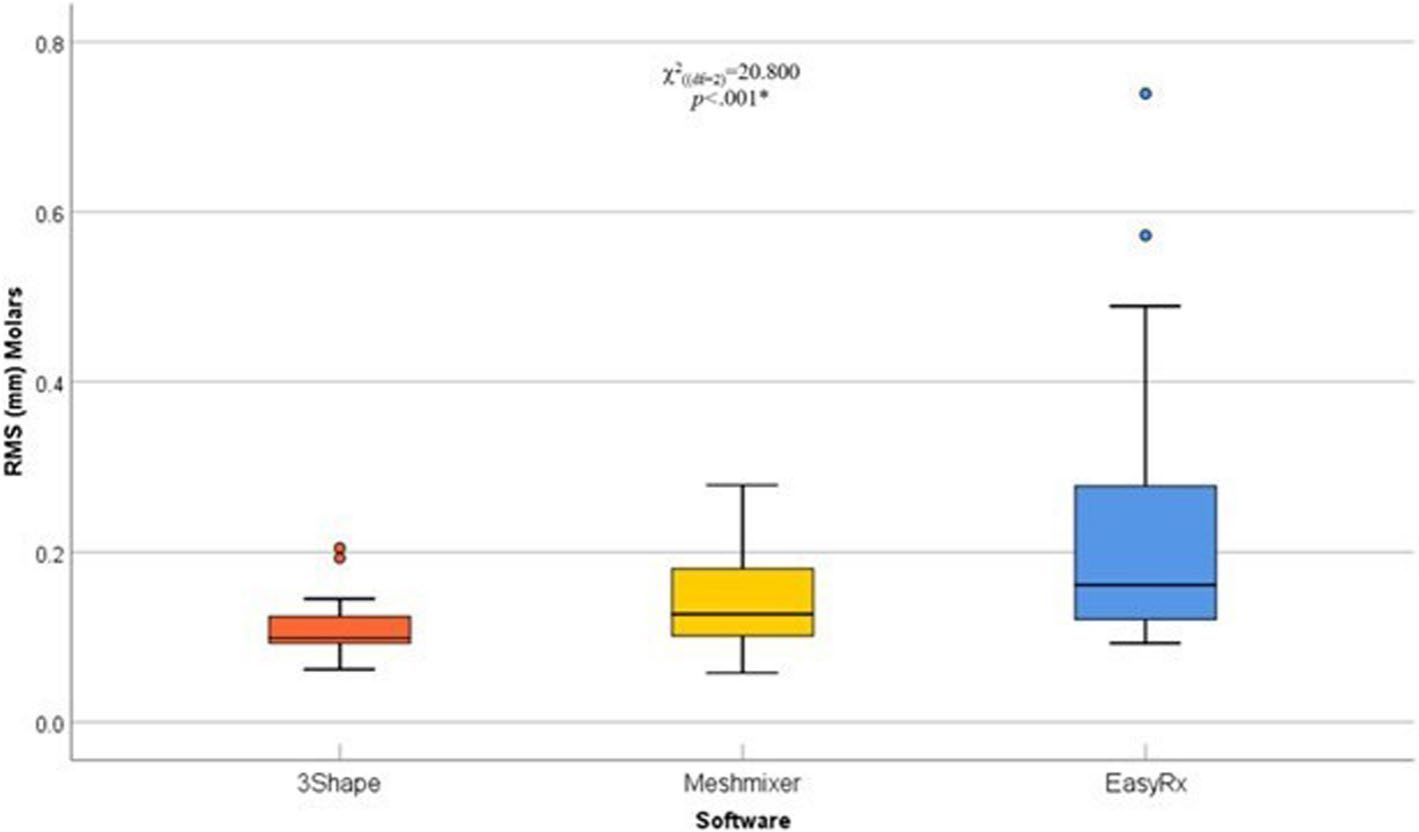
Box and whisker graph comparing RMS (mm) values in molars in the three different software, the thick line in the middle of the box represents the median, the box represents the inter-quartile range (from 25^th^ to 75^th^ percentiles), the whiskers represent the minimum and maximum

A pairwise comparison of the tooth segments (incisors, canines/premolars, and molars) in the three different software was done using averaged RMS surface changes. In incisors, surface changes by Meshmixer were significantly lower compared with 3Shape and EasyRx software (*p*=.03 and *p*=.006, respectively), the pattern of distribution is illustrated in (Table 2) (Fig. 7). Moreover, surface changes in canines/premolars by EasyRx were significantly higher compared with 3Shape and Meshmixer software (*p*<.001 and *p*=.001, respectively), the pattern of distribution is illustrated in (Table 2) (Fig. 8). Additionally, surface changes in molars by 3Shape were significantly lower compared with Meshmixer and EasyRx software (*p*=.005, and *p*<.001, respectively), the pattern of distribution is illustrated in (Table 2) (Fig. 9).

**Table 2:** Post-hoc pairwise comparisons between the three software used

	**Incisors**	**Canines and premolars**	**Molars**	**Overall**
	***P* value of post-hoc comparison**			
**3Shape vs. Meshmixer**	0.03*	1.00	0.005*	1.00
**3Shape vs. EasyRx**	1.00	<0.001*	<0.001*	<0.001*
**Meshmixer vs. EasyRx**	0.006*	0.001*	0.62	<0.001*

^*^Statistically significant differences between groups using Bonferroni adjusted significance level

**Fig. 10:**
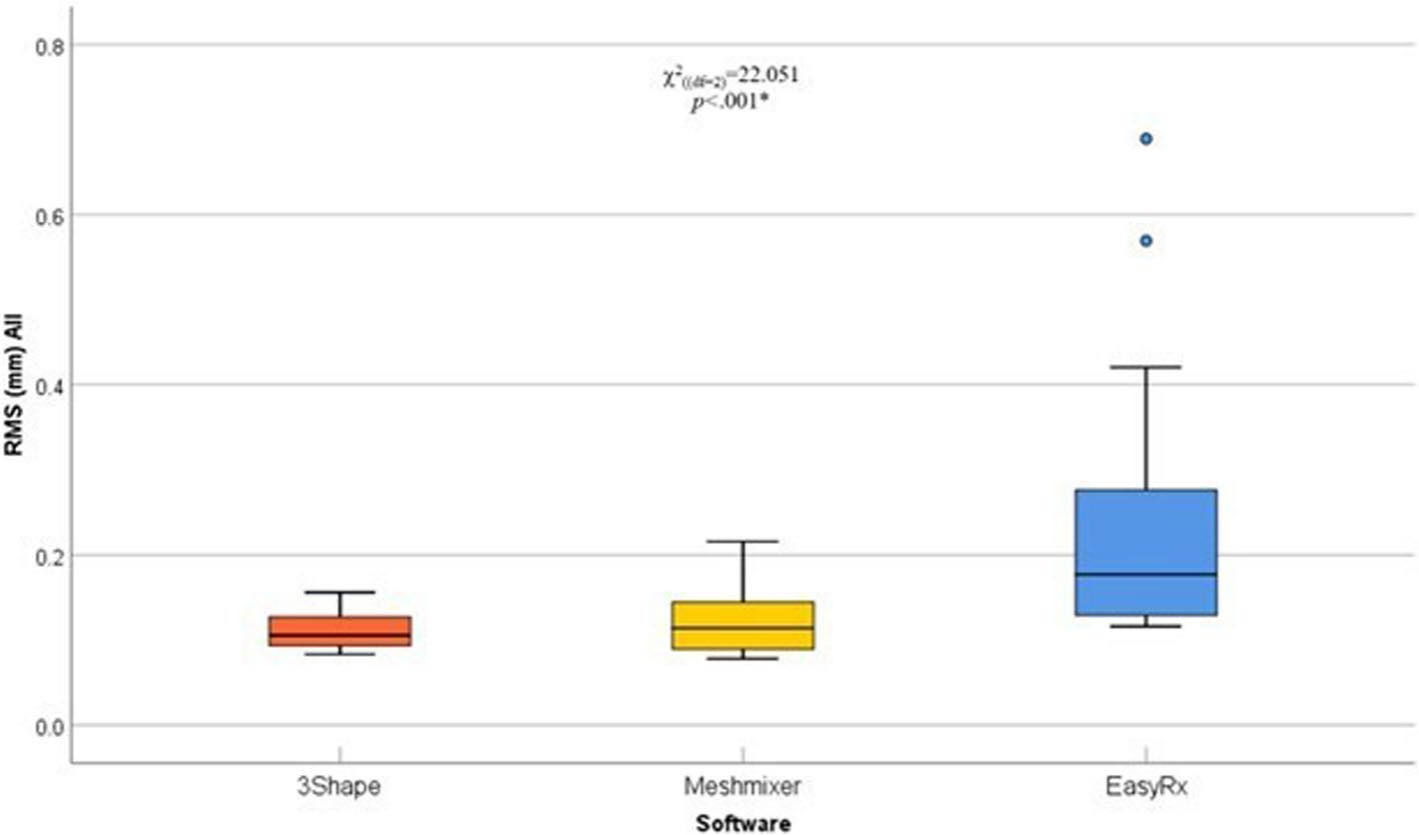
Box and whisker graph comparing RMS (mm) values in the overall model in the three different software, the thick line in the middle of the box represents the median, the box represents the inter-quartile range (from 25^th^ to 75^th^ percentiles), the whiskers represent the minimum and maximum

When evaluating the overall surface changes among the three different software, the Pairwise comparison of the RMS values of all segments combined demonstrated that EasyRx software had significantly higher values when compared to both 3Shape and Meshmixer software (*p*<.001 for all comparisons). The distribution pattern for each software is illustrated in (Table 2) (Fig. 10).

Positive and negative values were obtained for each segment. These data were then summed to estimate the percentage of positive and negative surface changes for each software, as shown in (Table 3). Among the whole sample of 720 teeth, it was seen that the positive results obtained from the three different software were significantly higher than the negative values.

**Table 3:** Comparisons of positive and negative tooth surface change after VBR

		**3Shape**	**Meshmixer**	**EasyRx**	**Total**	***P* value 1**
		**N (%)**				
**Incisors**	**Positive**	74 (92.50%)	68 (85.00%)	71 (88.75%)	213 (88.75%)	χ^2^= 2.25 *P*= 0.32
	**Negative**	6 (7.50%)	12 (15.00%)	9 (11.25%)	27 (11.25%)	
**Canines and premolars**	**Positive**	75 (62.50%)	74 (61.67%)	89 (74.17%)	238 (66.11%)	χ^2^= 5.23 *P*= 0.07
	**Negative**	45 (37.50%)	46 (38.33%)	31 (25.83%)	122 (33.89%)	
**Molars**	**Positive**	33 (82.50%)	38 (95.00%)	40 (100%)	111 (92.50%)	χ^2^= 19.47 P <0.001*
	**Negative**	7 (17.50%)	2 (5.00%)	0 (0%)	9 (7.50%)	
**Overall**	**Positive**	182 (75.83%)	180 (75%)	200 (83.33%)	562 (78.06%)	χ^2^= 5.90 *P*= 0.052
	**Negative**	58 (24.17%)	60 (25%)	40 (16.67%)	158 (21.94%)	
*P* value 2		χ^2^= 24.74 P <0.001*	χ^2^= 24.18 P <0.001*	χ^2^= 16.95 P <0.001*		

χ^2^: Chi-square test was used

*P* value 1: Comparison between the three study groups, *P* value 2: comparison between different segments within each group

^*^Statistically significant at *p* value <0.05

## Discussion

The reliability of commercially available intraoral scanners, as well as recent progressions in 3D printing and CAD/CAM software, has improved the feasibility of fabricating orthodontic appliances from 3D-printed models [23, 24]. It has been established before that using manual VBR software allowed prompt fabrication of orthodontic retainers [5]. Although AI offers a faster method of VBR, there is a lack of concrete evidence pertaining to its accuracy.

Through the VBR technique, buccal surfaces of the digital dentition can be reconstructed by the VBR software once the brackets and tubes are removed [18]. Previous research has demonstrated the accuracy of manual VBR procedures conducted through the utilization of 3Shape OrthoAnalyzer and Meshmixer software [5, 18]. In the current study, an evaluation and comparison were conducted on the aforementioned methodologies in relation to automated VBR performed by EasyRx software.

The CEREC Omnicam intra-oral scanner was utilized in this study as it possessed the necessary accuracy for such purposes [25–27]. Furthermore, its accuracy remained unaffected by the presence of orthodontic brackets, a crucial aspect that was given significant consideration in this particular study [28].

To assess the accuracy of VBR procedures, it was advisable to employ surface-based registration techniques to align digital models, followed by the computation of distances between surface points [19, 20, 29]. The accuracy of the 3D superimposition was crucial for the validity of any error analysis based on VBR, so a process of 3D surface-based superimposition of the pre-bonding and the post-bonding models was conducted as a first step using the Medit Link software. This would verify that changes identified on the labial surface of the teeth were exclusively attributed to VBR, rather than inaccuracies in the superimposition of digital models. The accuracy of the superimposition was verified by color-coded maps, ensuring that the registration error did not exceed a threshold of 0.05 mm [30, 31].

The decision to use averaged Root Mean Square (RMS) values rather than mean values was because RMS values encompass the overall magnitude of surface alterations, irrespective of the direction of change. Consequently, this approach offered a more comprehensive assessment of the changes.

Based on the results obtained from this study, it was shown that the accuracy of VBR exhibited a decline when transitioning from the anterior teeth to the posterior teeth. The least accurate VBR was evident in the molars segment, where the highest RMS values in the three tested software were observed. Conversely, the incisors in the three tested software exhibited the smallest RMS values. These findings suggest that the VBR technique was significantly less accurate in the posterior teeth.

Higher RMS values in the molars segment could be attributed to the close proximity of the posterior brackets to the gum line, which may have caused interference with the algorithm employed by the software to compute the target region and remove the bracket. In contrast, the positioning of brackets on the incisors was furthest from the gum line which might account for the better accuracy.

Moreover, it seemed that the algorithm of VBR was more accurate on flat surfaces such as incisors contrary to the curved surfaces of canines, premolars, and molars especially with the presence of grooves. This distinction was significant because it could have contributed to the higher margin of error in VBR procedures in molars, as the software utilized might have encountered challenges in accurately navigating the variations in surface curvature and in effectively removing the adhesive from within the buccal groove. Hence, the software reconstructed the anatomy of the molars with no buccal groove [5].

This was additionally translated by color mapping, revealing a greater prevalence of red hues in the molar segment, specifically in the groove area, which suggested a larger probability of insufficient bracket removal in the molar teeth as compared to the incisors.

The RMS values in the canines/premolars segment were found to be significantly lower compared to the molars for all software groups. This may be attributed to the fact that canines and premolars typically have a curved surface and brackets are usually positioned away from the gingival margins, in addition, there are no grooves on their surface. However, the results indicated that the RMS values for the canines/premolars segment were significantly higher in the EasyRx automated software group compared to the other two manual software groups. This suggested that the AI employed in the automated software might be less accurate than the manual technique in removing brackets from curved surfaces.

The accuracy of VBR using automated software was less than the ones using manual software. Despite the significant difference between EasyRx and both 3Shape and Meshmixer software, all changes caused by VBR remained within the reported 0.3-0.5 mm accuracy range for orthodontic models [32]. Therefore, the surface changes may not possess clinical significance.

It was observed that all three groups exhibited a significantly higher percentage of insufficient bracket removal than unintentional tooth surface removal. Clinically, in orthodontic retainers, the effect of minor insufficient bracket removal of less than 0.3 mm might be preferable to the unintended removal of the tooth surface. Insufficient removal of brackets would be associated with a lower likelihood of difficulties with the insertion of orthodontic appliances.

Although the unintentional removal of tooth surfaces might enhance the appliance fit, it also runs the risk of being too active against the tooth, with potential resultant discomfort and /or inadvertent tooth movement. However, in case of insufficient bracket removal of larger magnitudes, the greater distance between the dentition and retainer increases the possibility of relapse, as a result of a poor fit [33].

In clear aligners, this discrepancy of fit could be a potential cause for the lack of accuracy in some planned orthodontic movements, resulting in the failure of teeth to follow their planned movement and with subsequent need for refinements [34].

The findings of our study were consistent with those reported by Marsh et al. [5]. Their study demonstrated higher RMS values in the posterior teeth compared to the anterior teeth with the first molar segment exhibiting the highest values of surface changes. In terms of the direction of surface changes, the present study revealed a similar distribution pattern, with a higher percentage of positive surface changes observed after VBR in all tooth segments. In contrast, the Chamberlain-Umanoff study [18] revealed a negative direction of change in the incisors and molars segments, suggesting unintentional tooth surface removal. Nevertheless, it demonstrated a positive direction of change in the canines/premolars segment.

The observed variations may be attributed to the difference in the methodology used in the typodont study [18], which employed in vitro experimentation with 3D printed resin models. On the other hand, our study and the study conducted by Marsh et al. [5], utilized in vivo investigation on actual tooth surfaces.

Given that the difference in the accuracy between the three software was deemed minimal and of negligible clinical significance, there are additional considerations that may influence the selection of the preferred VBR approach, including cost, VBR technique, and the duration of the VBR process (Table 4).

**Table 4:** Comparison between 3Shape, Meshmixer and EasyRx software

**Factor**	**3Shape**	**Meshmixer**	**EasyRx**
Cost	Commercially available	Free	Commercially available
Technique	Manual	Manual	Automated
Time	10-15 mins/arch	10-15 mins/arch	~ 5 mins/arch

One limitation of this study was the inability to measure the fit of appliances fabricated from models obtained after VBR. As a result, future research is recommended using post-debonding cases to assess the fit of appliances fabricated from 3D-printed models after VBR.

## Conclusions

- 3Shape and Meshmixer manual VBR software were found to be more accurate than EasyRx automated software, however, the differences were minimal and clinically insignificant.
- The three tested software showed insufficient bracket removal after VBR across all teeth segments.
- The three tested software performed less accurately on curved surfaces than flat surfaces, and the VBR process might be complicated by the presence of grooves.

## Data Availability

The datasets used during the current study are available from the corresponding author onreasonable request.

## Abbreviations

VBR: Virtual bracket removal
CAD: Computer-aided design
CAM: Computer-aided manufacturing
STL: Stereolithography
RMS: Root mean square
AI: Artificial intelligence
